# Proteome Analysis of *Nicotiana tabacum* Cells following Isonitrosoacetophenone Treatment Reveals Defence-Related Responses Associated with Priming

**DOI:** 10.3390/plants12051137

**Published:** 2023-03-02

**Authors:** Nikita da Camara, Ian A. Dubery, Lizelle A. Piater

**Affiliations:** Department of Biochemistry, University of Johannesburg, Auckland Park 2006, Johannesburg P.O. Box 524, South Africa

**Keywords:** defence, electrophoresis, isonitrosoacetophenone, iTRAQ, plant cells, priming, proteome

## Abstract

Proteins play an essential regulatory role in the innate immune response of host plants following elicitation by either biotic or abiotic stresses. Isonitrosoacetophenone (INAP), an unusual oxime-containing stress metabolite, has been investigated as a chemical inducer of plant defence responses. Both transcriptomic and metabolomic studies of various INAP-treated plant systems have provided substantial insight into this compound’s defence-inducing and priming capabilities. To complement previous ‘omics’ work in this regard, a proteomic approach of time-dependent responses to INAP was followed. As such, *Nicotiana tabacum* (*N. tabacum*) cell suspensions were induced with INAP and changes monitored over a 24-h period. Protein isolation and proteome analysis at 0, 8, 16 and 24 h post-treatment were performed using two-dimensional electrophoresis followed by the gel-free eight-plex isobaric tags for relative and absolute quantitation (iTRAQ) based on liquid chromatography and mass spectrometry. Of the identified differentially abundant proteins, 125 were determined to be significant and further investigated. INAP treatment elicited changes to the proteome that affected proteins from a wide range of functional categories: defence, biosynthesis, transport, DNA and transcription, metabolism and energy, translation and signalling and response regulation. The possible roles of the differentially synthesised proteins in these functional classes are discussed. Results indicate up-regulated defence-related activity within the investigated time period, further highlighting a role for proteomic changes in priming as induced by INAP treatment.

## 1. Introduction

The exposure of plants to the various biotic or abiotic stressors present in the surrounding environment results in activation of the necessary defence mechanisms required for survival [1]. The lack of an adaptive immune system forces plants to rely upon an inherent two-tier defence strategy composed of both preformed and inducible innate immune systems. Multi-layered cellular and molecular networks are involved in effective adaptation to changing environments. Here, integrated processes such as effective perception, signalling cascades, cell wall strengthening, synthesis of defence-related proteins and deployment of anti-microbial metabolites contribute to an effective defence [2]. 

Successful host survival following stress exposure results in an enhanced basal resistance capacity, allowing for a more rapid response to future attacks through broad spectrum defences, primarily systemic acquired resistance (SAR) and induced systemic resistance (ISR) [3,4,5]. These defences may be similarly triggered by various chemical agents. Examples of these include β-aminobutyric acid (BABA) as well as salicylic acid (SA) and its functional analogues 2,6-dichloro-isonicotinic acid (INA) and benzo 1,2,3-thiadiazole (BTH) [3,4]. Utilisation of chemical-based elicitation of plant defences is a part of the priming phenomenon, defined as the pre-exposure of host plants to defence-inducing stimuli, mimicking biotic and abiotic stressors and allowing plant adaptation in launching defence responses more rapidly [6]. This stimulation of the immune system of plants could be developed as alternative strategies that hold potential for enhancing the capacity of plants to cope with biotic as well as abiotic stressors. The majority of the studies regarding characterising and defining the biochemical changes related to priming processes have been driven by targeted methods. These approaches have played a vital role in clarifying the main features of priming, including enhanced perception systems, dormant signal transduction enzymes, phytohormones, transcription factors and chromatin modification. However, there are still gaps in our general knowledge concerning the dynamism and complexity of molecular mechanisms involved in the entire priming event, considering the complexity of multi-layered biological information networks [6].

Despite our relatively detailed knowledge of mechanisms of the plant innate immune system, many aspects of the response to pathogen attacks remain uncharacterised. Previous biochemical studies of the mechanism of action of the chemical inducer, isonitrosoacetophenone (INAP), in *Nicotiana tabacum* (*N. tabacum*) cell suspensions reported the production of secondary metabolites involved in plant–microbe interactions as well as triggering of pathways associated with the defence response [7]. Further transcriptomic studies indicated the expression of multiple genes linked to pathogen perception, signal transduction and processing and the underlying biochemistry of defence responses [8]. However, how these transcriptional changes reflect the remodelling at the proteome level is unclear.

In the present study, isobaric tags for relative and absolute quantitation (iTRAQ) was utilised to determine proteomic changes following INAP treatment of *N. tabacum* cell suspensions as a continuation of previous work in validating this oxime-containing chemical as an inducer of priming. In total, 1530 proteins, including 10 decoys (Table S3), were positively annotated, among which 125 displayed differential regulation of note. Analyses of these proteins revealed several pathways associated with a triggered defence response and allowed for correlation of INAP-associated responses with previously conducted studies.

## 2. Results

### 2.1. Two-Dimensional Electrophoresis

**Figure 1:**
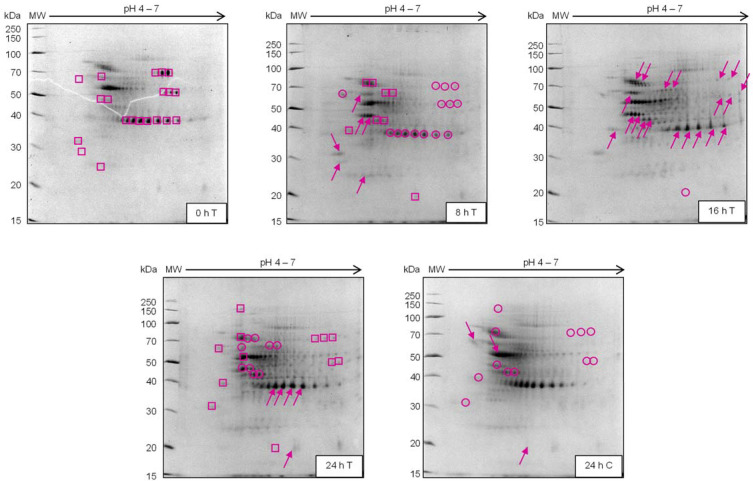
Representative narrow range (pH 4–7) 10% SDS-PAGE two-dimensional gels using 100 µg of protein isolated from *N. tabacum* cells treated with 1 mM isonitrosoacetophenone (INAP). Comparative analysis was performed using 0 h as the reference state and each time point thereafter juxtaposed to the previous time point. C, control; T, treated; squares, initial protein state; arrows, up-regulation; circles, down-regulation.

Analysis of the *N. tabacum* samples was conducted utilising a side-by-side semi-quantitative comparative approach to efficiently track proteins of differential abundance through various time points (from 0 h to 24 h) (Figure 1).

At each time point, analysis was done in comparison to the previous, thereby revealing proteins of significance and correlated to INAP treatment. Included was the 24 h control (C) sample, considered a direct comparison for proteomic variation due to INAP treatment when juxtaposed against the 24 h treated (T) sample, and an added comparison of cellular changes during 0–24 h. Visual inspection of the two-dimensional electrophoresis (2-DE) protein maps indicated up-regulation from 0 h to 8 h, as well as from 8 h to 16 h, followed by a high degree of down-regulation from 16 to 24 h. The greatest degree of up-regulation was present within the 16 h sample while the 24 h sample showed the greatest degree of down-regulation. Further comparison of the biological repeats displayed consistency with respect to the regulated states at the various time points while the protein maps remained distinctive from one time point to the next.

Comparison of the 24 h T to the 24 h C indicated the down-regulation of several proteins following INAP treatment. Therefore, the treatment of *N. tabacum* using INAP induced proteomic effects lasting 24 h, with the significant proteomic changes occurring at 16 h.

### 2.2. iTRAQ LC-MS Analysis

**Figure 2:**
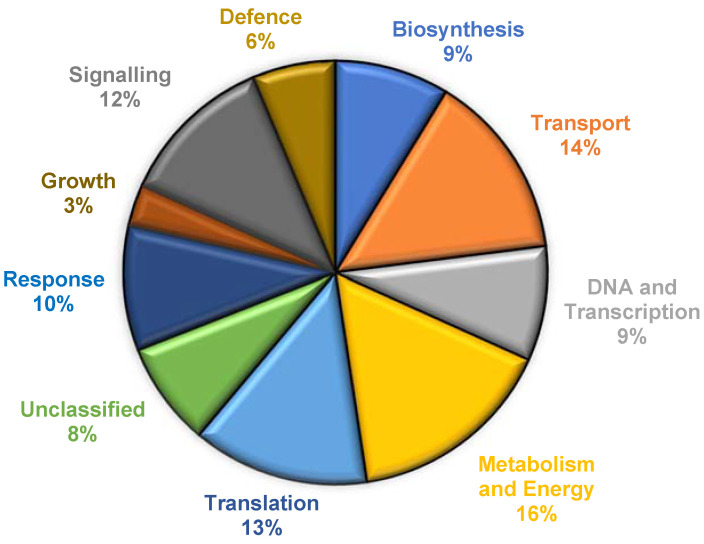
Functional classification of INAP-responsive proteins identified through iTRAQ. The chart represents proteins identified from *N. tabacum* cell suspensions across the time study of 0–24 h, expressed as percentages, and determined in relation to the reference/control (0 h). Functional category terms were obtained from Uniprot and confirmed following TBLASTN and the available literature [8].

The total complement of proteins found to respond differentially towards INAP treatment are listed according to subcellular location in Table S1 (cytosolic, 100 proteins) and Table S2 (chloroplast, mitochondria and ribosomes, 25 proteins). Analysis was performed to determine potential significant trends amongst the proteins attributed to INAP-induced responses and these proteins were categorised according to the functional categories (Figure 2). The 48 key proteins displaying differential/significant regulation are listed in Table 1. Uncharacterised proteins are referred to by accession number followed by the identified gene where applicable. In addition, some identified proteins might belong to more than one category, e.g., ‘biosynthesis’ and ‘defence’.

Following analysis of the response/adaption-related and basic functionality proteins of significance following INAP treatment, stacked bar graphs were constructed for the INAP-treated samples. From these graphs, we determined which time point(s) were the most crucial and the overall processes affected subsequent to INAP exposure. It should be noted that when specific processes or proteins are referred to, the overview of the functional category should not overshadow the specific up- or down-regulated state of the individual protein as discussed under the relevant process as the graphs encompass all the proteins including those not discussed.

## 3. Discussion

Following a priming event/inducer treatment, plants become cellularly and organismally reprogrammed in a long-lasting manner, with a ‘memory’ of such events at a molecular level. Depending on the initial stimulus and the target of priming, primed plants can deploy a diverse set of defence mechanisms that are more rapid and stronger compared with non-primed plants [9]. A metabolomic analysis of INAP-induced responses in *N. tabacum* cells has indicated major changes in hydroxycinnamic acids and other phenylpropanoids due to its probable recognition by the enzymatic machinery of the cinnamic acid pathway [7]. Accumulation of free phenolics can have detrimental effects on cell viability but can be mitigated through the formation of ester and amide conjugation and vacuolar storage [7]. A subsequent contribution presented evidence that INAP drives transcriptional reprogramming in these cells in support of innate immunity and defence [8]. In the absence of a pathogenic threat and in order to re-establish cytosolic equilibrium, triggered responses need to be down-regulated. Here, we report on the proteome changes, using 2-DE and iTRAQ technologies, resulting from INAP treatment with a focus on priming and establishing a defensive capacity in the cells. While 2-DE provided a comprehensive, visual map of changes occurring at the proteome level, the iTRAQ procedure was used since it is able to identify more proteins at the peptide level, even proteins with a low copy number, membrane proteins, or other proteins that are difficult to detect by 2-DE.

### 3.1. Adaption-Related Proteins Responsive to INAP Treatment

Of the identified differentially abundant proteins related to the INAP treatment of *N. tabacum* cell cultures, 125 were determined to be of interest and were further investigated. An overview of the adaption-related proteins indicates that the affected processes are primarily related to cellular signalling, cell wall enhancement, anti-microbial response generation and growth limitation/reduction. It was determined that, in general, these pathways were active at 8 h and deactivated at 16 h as a down-regulation of the original trigger event.

#### 3.1.1. Biosynthesis and Redirection of Resources

**Figure 3:**
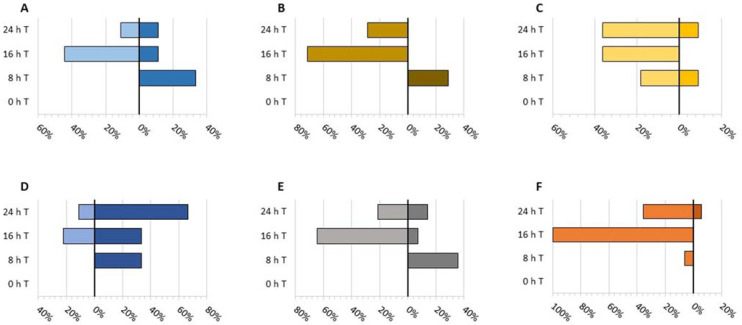
Stacked bar graphs of the adaption-related proteins for the determination of crucial time points following INAP treatment in *N. tabacum* cell suspensions. The graphs were created with respect to the prominent functional categories: (**A**) biosynthesis, (**B**) defence, (**C**) metabolism and energy, (**D**) response, (**E**) signalling and (**F**) transport. The percentages were determined through calculation of the number of either up- or down-regulated proteins relative to the number of proteins within the respective categories. Values to the left of zero (0) indicate down-regulation while those to the right depict up-regulation. The colour code is as for Figure 2.

The involvement of biosynthesis-related proteins in response to the triggering of priming (defence) events plays a crucial role in the redirection of resources for the establishment of applicable survival countermeasures. These processes range from ATP generation to drive the synthesis of defence-associated metabolites and lignin synthesis for cell wall reinforcement to the synthesis of the necessary amino acids required for the production of defence proteins and precursors of secondary metabolites [9]. The observed up/down-regulation of these proteins as seen in Figure 3A indicates that the majority of the biosynthetic responses to INAP treatment happen prior to 24 h.

Analysis of the biosynthesis proteins (Table S1) showed intriguing differences between the control and INAP-treated samples. Of these, the most significant included M1C547_SOLTU (isopentenyl-diphosphate delta-isomerase I). This enzyme, a multi-isoform protein in plant species, is responsible for the first step in terpenoid biosynthesis [10] and the related protein showed increased levels at both 8 and 24 h. Terpene/terpenoid molecules are utilised amongst anti-microbial metabolites in preformed and induced immunity [11], thus suggesting anti-microbial generation in response to INAP elicitation [7].

Two UPD-linked proteins, involved in processes related to the cell wall, were identified: M1C5Y4_SOLTU (UDP-glucuronic acid decarboxylase 6-like) and SlUPTG1 (UDP-glucose:protein transglucosylase-like protein). The former (up-regulated at 8 h) is associated with the glycosylation of caffeic acid, a key precursor metabolite for lignin biosynthesis through the phenylpropanoid pathway [12], and was previously linked to INAP treatment [8], while the latter (a slight change at 8 h/unchanged) is necessary for the biosynthesis of hemicellulose/glycoprotein precursor production for both primary and secondary cell walls [13]. Both proteins indicate preparation for cell wall reinforcement, a process typically aimed at pathogen restriction [14]. However, changes in cell wall composition may also be attributed to priming, as seen in SA-primed maize hybrid seeds where an adjustment of cell wall elasticity was necessary for chilling tolerance [15].

Down-regulated proteins included K4BVZ4_SOLLC (dolichyl-diphospho-oligosaccharide-protein glycosyltransferase), K4CET6_SOLLC (long chain acyl-CoA synthetase 8-like) and cystathionine gamma-synthase. The first, responsible for lipid/protein glycosylation, often serves as an indication of infection, where a lack thereof frequently results in disease [16]. In *Arabidopsis thaliana*, long-chain acyl-CoA synthetase activates C_16_ and C_18_ long-chain fatty acids for the synthesis of cuticular wax and is well-defined in fatty acid transport and lipid metabolism [17]. This protein also provides acyl-CoA for N-myristoylation, which is important for signal transduction pathway components and suggests a decrease in lipid metabolism but may serve as an indication of potential lipid signalling [18]. Furthermore, cystathionine gamma-synthase, the first committed step in methionine biosynthesis, was down-regulated, an occurrence seen previously at the transcriptomic level by Zhou et al. (2007) in salt-stressed tomatoes [19].

#### 3.1.2. Defence

Induction of defence responses in plants requires the utilisation of proteins specialised in the perception of pathogen presence and triggering of the necessary downstream responses such as phenylpropanoid biosynthesis [7,20]. The most rapid defence response involves the onsite production of reactive oxygen species (ROS) subsequent to a pathogen attack [5]. Another major contributor to plant defences is the cell wall, undergoing ROS-dependent dynamic changes for the prevention of pathogen incursion [21].

Two proteins of differential abundance, up-regulated in the 8 h treated sample (Table 1), were annexin and a putative hydroxycinnamoyl transferase. The former, a calcium-dependent phospholipid-binding protein, is suggested to play a role either directly or indirectly in the oxidative stress response [22], while Jami et al. [23] reported the increased tolerance of transgenic tobacco to biotic and abiotic stressors through ectopic annexin expression from *Brassica juncea*. Hydroxycinnamoyl transferase, identified as shikimate O-hydroxycinnamoyltransferase, constitutes a part of several enzymes involved in monolignol biosynthesis [24]. Polymerisation of these precursors results in the synthesis of lignin and suberin isoforms for cell wall strengthening [7]. Both of the aforementioned proteins have been shown to be induced under application of the defence-associated phytohormone abscisic acid (ABA) [25]. Furthermore, these proteins, in conjunction with the UDP-linked proteins, strongly suggest INAP-induced cell wall reinforcement and maintenance occurring at around 8 h, a large contributor to the up-regulation seen in Figure 3A and 3B, thus supporting a primed state by enhancement of basal resistance [3].

Conversely, superoxide dismutase (Cu-Zn), polygalacturonase inhibiting protein (PGIP) and K4ASK1_SOLLC (callose synthase 9-like) (Table 1) were found to be only differentially regulated (down-regulated) in the 16 h treated sample. The defence-associated PGIP, for the limitation of fungal polygalacturonases, is a component of plant innate immunity [26]. PGIPs show altered expression in accordance with elicitation type, i.e., biotic or abiotic, and hence are priming agents for the onset of SAR [1]. The transport of callose synthase to its site of requirement during defence has previously been reported to be a positive response protein under abscisic acid (ABA) and beta-aminobutyric acid (BABA) priming against necrotrophic pathogens [27]. However, its interference with fellow proteins associated with cell wall integrity suggests that others, such as the UDP-linked biosynthesis proteins, are prioritised for lignin biosynthesis versus callose formation [28]. Furthermore, callose synthase activity was reported to be modulated under stress conditions by annexin [22], a protein seen to be up-regulated in cucumber by the chemical inducer acibenzolar-*S*-methyl (ASM) [29].

#### 3.1.3. DNA and Transcription

Differential gene expression and transcription form two vital components of the defence response, affecting proteomic changes for downstream defence applications. The apparent down-regulation of proteins K4CMV1_SOLLC (replication protein A 70 kDa DNA-binding) and endonuclease 1 (Table 1), as proteins involved in replication and repair, suggests that treatment of *N. tabacum* cells with INAP reduces chromosomal expansion at later time points [30]. Of particular interest is the decline in protein levels at 16 h of histone H2B, a fundamental chromatin element typically associated with viral infections of host plants where it participates in chromatin modification that results in a decrease in transcription. These observations might reflect a redirection of gene transcription towards increasing basal resistance [8].

#### 3.1.4. Growth, Metabolism and Energy

Plant growth is optimal when unhindered by stressors. However, plants are generally in either a growth or a defensive state, and a trade-off must be made with regard to the allocation of resources [20,31]. *N. tabacum* proteins DWARF1/DIMINUTO and K4BLX1_SOLLC (BONZAI 1-like) (Table 1), both associated with the plasma membrane during cell division, were down-regulated at 16 h post-treatment, thus indicating a potential negative effect on cellular growth due to INAP. The first protein, an α-subunit of G protein, is linked to signal transduction pathways controlling cell division and differentiation [32]. BON1 in vitro promotes the aggregation of lipid vesicles, and its loss may lead to a reduction in cell division and expansion [31]. However, the decreased levels of the two abovementioned proteins were found to occur only in the 16 h treated sample. The return to levels comparable to 0 h in the 24 h treated sample might be an indication of re-establishment of homeostasis.

Differential up-regulation of K4BGV0_SOLLC (CDK5RAP3-like protein) (Table 1) supports a reduction in growth through its inhibition of cyclin D1 expression, a requirement for G1/S transition [33]. Furthermore, this protein, in combination with the other observed growth effector proteins, most likely promotes a transient decrease in *N. tabacum* cellular growth due to the redirection of energy reserves towards a primed or defensive state.

Redirection of resources to defence pathways requires utilisation of the primary metabolism for the promotion of defence [7]. Accordingly, not all metabolic processes remain unaffected, e.g., photosynthesis has been reported to be down-regulated during pathogen infection, and a high degree of down-regulation can be observed at several of the time points in Figure 3C. Adaptive stress metabolism therefore plays a significant role in plants that are experiencing stress [7,9]. The down-regulation of the cell-wall-related K4BDG0_SOLLC (very-long-chain 3-oxoacyl-CoA reductase 1-like) and M1A251_SOLTU (suberisation-associated anionic peroxidase 1-like) in the 16 and 24 h INAP-treated cells (Table 1) suggests that cutin biosynthesis and suberisation processes are not prioritised [34]. This is further supported by the up-regulation of lignin-biosynthesis-associated proteins at 8 h (Figure 3A–C) and the down-regulation of these proteins at 16 h, including the long-chain acyl-CoA synthetase. In addition, the defence protein hydroxycinnamoyl transferase (Table 1), triggered at 8 h, increases the likelihood that cell wall/membrane processes will be completed between 8 and 16 h. Probable pectate lyase P18 (Table 1), another cell-wall-related protein, was unchanged following INAP induction. Active pectate lyases, in combination with polygalacturonases, trigger cellular growth via cell wall loosening but, when deactivated, result in a growth reduction and apical wall stiffening [35]. These cell wall responses, in particular lignification, are mechanistically associated with SAR and ISR and may contribute to an enhanced defensive state as would occur in primed plants [36].

Other proteins of differential abundance, at various time points but primarily at 8 h and 24 h, are the K4AT35_SOLLC (26S proteasome non-ATPase), glycylpeptide N-tetradecanoyltransferase and methylenetetrahydrofolate reductase proteins (Table 1). The majority of protein degradation occurs through the 26S proteasome in association with ubiquitin, a system with documented roles in hormone signalling and disease resistance, the inhibition of which results in suppression of host defences [8]. The unchanging levels of glycylpeptide N-tetradecanoyltransferase for the given time points indicate the presence of N-myristoylation and lipid metabolism, resulting in possible post-translational protein modification as observed in salt-stressed tomato [19]. Therefore, the previously mentioned decrease in long-chain acyl-CoA synthetase indicates that while cutin biosynthesis was decreased, lipid signalling may be associated with INAP treatment. An increase in methylenetetrahydrofolate reductase (a rate-limiting enzyme in the methyl cycle, responsible for maintaining the methyl pool required for methylation of both DNA and protein) was noted in responses of both heavy-metal-stressed tobacco roots and elicitor-induced rice cells as a jasmonic acid (JA)-responsive gene [37]. This protein was also reduced in *Actinidia chinensis* when infected with *Pseudomonas syringae* [38]. However, the up-regulation at 8 h in *N. tabacum* cells following exposure to INAP would suggest a positive influence for the production of the necessary amino acids required for the synthesis of new proteins involved in priming or defence.

#### 3.1.5. Stress-Related Responses and Signalling Events

Dynamic environmental changes allow for various interactions between plants and their surroundings and may result in both biotic and abiotic stresses. The ability to activate an appropriate response necessary to ensure plant survival relies upon protein activity often associated with perception of the stressor [20]. *N. tabacum* proteins listed in this category (Table 1) include glycine-rich RNA-binding protein and putative methyltransferase. The results indicate an increase in protein levels (Figure 3D) within INAP-treated cells harvested at several of the time points. Glycine-rich RNA-binding protein, associated with the cell wall, was up-regulated at a later time point (24 h) as also observed pertaining to INAP [8]. The expression of these proteins is linked to external stressors such as cold, salinity, wounding and viral infection [39].

Some of the down-regulated proteins detected were methyltransferases (e.g., down-regulated in the 16 h treated sample). These include a wide array of methyl-transferring proteins, such as caffeoyl-CoA-*O*-methyltransferases and serine hydroxymethyltransferase, that affect monolignol synthesis [8]. However, previous proteins detected amongst the biosynthesis and defence categories suggest that these methyltransferases are not linked to lignin biosynthesis.

Elicitor-triggered signalling has a degree of overlap between P/MAMP-triggered immunity (P/MTI) and effector-triggered immunity (ETI) activation or repression through the mediation of signalling hormones that include SA and JA for the triggering of defence responses [37]. Other key players in plant signalling are found in the form of protein kinases that orchestrate responses from stimuli [40]. Signalling proteins of differential abundance (Table 1) include, among others, Ras-related proteins and protein kinases. The former protein superfamily comprises five subfamilies, of which the only signalling proteins are the Ras and Rho GTPases [41]. These have been implicated in signal transduction of hormonal/sensory signals across the plasma membrane, while superfamily-relative Rab proteins regulate vesicle targeting on the cytoplasmic side of the plasma membrane [42]. Down-regulation of the Ras protein suggests an avoidance of cell death due to its observed positive regulatory role of hypersensitive response (HR)-mediated programmed cell death (PCD) in *A. thaliana* [8]. On the other hand, the down-regulation of K4BTJ3_SOLLC (Rab 7) indicates a decline in vesicle transport linked to degradation [42].

Additional INAP-responsive proteins related to signal transduction were M1B9Y5_SOLTU (inactive receptor kinase) and calcium-dependent protein kinase (CDPK). Up-regulation of CDPK has previously been reported in relation to INAP treatment [8]. CDPK triggered by stress was reported to be a contributor of early responses to both biotic and abiotic stressors [43]. However, CDPKs have been additionally implicated in an *Avr*-specific HR, in which case down-regulation as observed in conjunction with the decrease in the Ras proteins would ensure prevention of the HR [8]. Furthermore, the return of all of the aforementioned proteins to levels relative to those seen at 0 h indicates a recycling of these signalling counterparts suggesting that the majority of protein signalling events occur prior to 16 h, resulting in the high degree of down-regulation in Figure 3E.

Other proteins induced following INAP treatment include beta-tubulin, the microtubule constituent, and the central metabolic and defence regulator SnRK1 [40,44]. Alpha-tubulin activation has previously been reported in response to INAP [8], while both alpha- and beta-tubulin have been recorded in the *A. thaliana* response to *Plasmodiophora brassicae* [45]. Gerber et al. (2008) [40] further indicated the convergence of signalling upon cytoskeletal proteins during crucial processes such as vesicle trafficking. The up-regulated SNF1-related protein kinase 1 (SnRK1) at 8 h provides a close relationship between metabolism and defence, although it serves as a metabolic regulator [46]. SnRK1 has previously been associated with a range of other processes, including stress hormone signalling, HR and acclimation against pathogens such as geminiviruses [44]. Therefore, these proteins serve as central signalling hubs linked to several pathways for adequate responses [44] and, in relation to this study, responses to INAP. Another protein displaying variable differentiation under INAP treatment is phospholipase D (PLD), which was up-regulated at 8 h but down-regulated at 16 h. Together with phosphatidic acid, PLD plays a role in various plant defence responses (from protein–lipid and protein–protein interactions to hormone signalling) [47]. The regulatory roles of PLDs in plants include ABA signalling, PCD and other stress responses, and PLD itself is directly regulated by heterotrimeric G proteins at the plasma membrane [48]. The differential regulation of PLD indicates that lipid signalling, as promoted by other identified proteins, is activated in response to INAP treatment, with a potential convergence of signalling at the microtubules [49].

#### 3.1.6. Translation

An effective plant defence in response to pathogen incursion and, by proxy, priming requires not only cell wall reinforcement and phytoalexin production but also the de novo synthesis of defence proteins [50] for effective and timeous defence responses [8]. A translation-associated protein of interest was found in the chaperone protein peptidyl-prolyl cis-trans isomerase (PPIase) listed in Table 1. PPIases catalyse the isomerisation of proline residue peptide bonds whilst facilitating de novo protein synthesis, reactivation of denatured proteins and the refolding of wound-damaged proteins [8,51]. The up-regulation of PPIase and the unchanged DnaJ-like chaperone protein thus suggest potential protein synthesis and maintenance in response to INAP. Down-regulation of several ribosomal proteins (Table S2) in INAP-treated cells might indicate a decrease in general protein synthesis, such that low levels are maintained only for the production of necessary INAP-response-related proteins [8].

#### 3.1.7. Transport

Transport in plants plays a crucial role, not just in homeostatic functioning but in the translocation of proteins towards an appropriate cellular location. In addition, transport is required for the successful secretion of antimicrobial compounds associated with the defence response [52]. Analyses of the transport proteins distinctly affected by INAP in Table 1 show a large number of proteins displaying down-regulation, primarily in the 16 h treated sample (Figure 3F). This may serve as an indication of the after-effects of responses triggered at the earlier time points by INAP. Proteins such as the clathrin heavy chain are required for receptor-mediated endocytosis and are also involved in receptor turnover at the plasma membrane [53]. The transport of protons and heavy metals, such as ATPase4 and M1D6E0_SOLTU (ATPase 2), respectively, is linked to the transport of Zn^2+^ from the roots to shoots in *A. thaliana* and has been reported to be influential in mitigating salt stress [54]. The potential difference across the membrane generated by ATPase 4 is required by the secondary transporter activity involved in organic compound transport [55]. Pleiotropic drug resistance proteins have been highlighted in the JA-triggered defence response with roles in antimicrobial terpene secretion following biotic stresses [52]. The COPII-associated proteins, reticulon-like protein and K4CIM3_SOLLC (Protein transport protein SEC16B), were both down-regulated at 16 h and found at the endoplasmic reticulum [56]. Reticulons function as membrane stabilisers while SEC16 defines the ER region for COPII complex assembly [57]. SEC12, another ER membrane protein required for COPII complex assembly, is responsible for the activation of the GTPase Scar1, a signalling protein identified in the *N. tabacum* INAP-treated transcriptomic study [8].

### 3.2. Basic Functionality Proteins

Basic functionality proteins or ‘housekeeping’ proteins, which include plastidic, ribosomal and mitochondrial proteins, typically function in normal cellular metabolism and upkeep but may be redeployed when exposed to stressors or patho-physiological conditions, thereby resulting in altered protein patterns [58]. This shift from housekeeping to defence metabolism is triggered by altered regulatory and signalling circuits and from amplified demands for energy and biosynthetic capacity. The up-regulation of this class of proteins, such as those closely related to primary metabolism (Figure 3B), thought to aid defence processes, was observed for several of the *N. tabacum* proteins listed in Table 1. This is supported by the differential regulation of proteins in the ‘Metabolism’ category (glycine dehydrogenase, aspartate aminotransferase and K4C412_SOLLC (fumarate hydratase 1)) [59,60]. These changes might affect nitrogen (aspartate) and carbon (oxaloacetate) metabolism and link the associated metabolic cycles in support of anaplerotic reactions to replenish the citric acid cycle if it becomes depleted of intermediates by biosynthetic demands. Furthermore, alterations in carboxylic acids levels were reported to be perceived in plants during stress responses and it was suggested that the tricarboxylates could modulate signal transduction cascades linked to plant defence responses [61]. In this context, citrate and fumarate were reported to be inducers of defence priming through complex signalling pathways in *A. thaliana* [61].

#### Mitochondrial Activity

**Figure 4:**
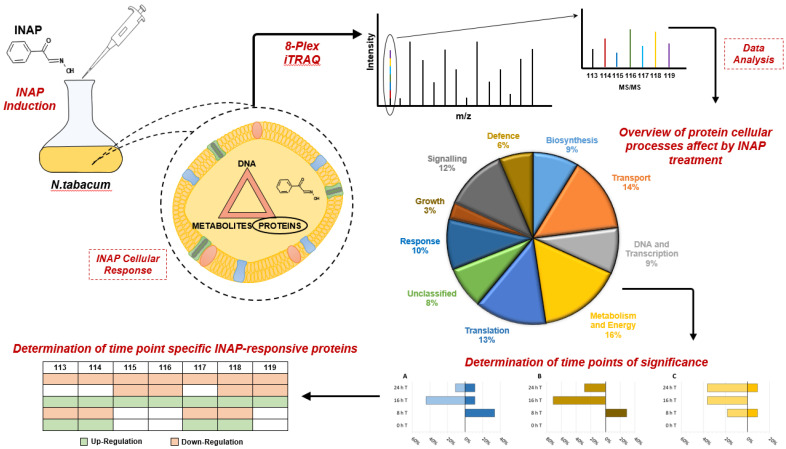
A graphical abstract indicating the proteome processes affected by *N. tabacum* cellular treatment with INAP.

Down-regulation of both the mitochondrial cytochrome c and a small heat shock protein may be due to its association with mitochondrial activity [62]. Plant mitochondria act as central hubs in plant metabolism, are integrated into cellular responses to environmental challenges and contribute to cellular homeostasis through redox balancing and retrograde signalling [63]. Relatedly, the down-regulation of another mitochondrial protein, cytochrome P450, at 16 h suggests that the processes it is involved in, namely plant hormone (JA) and phytoalexin biosynthesis, have been brought to completion [8,37]. In summary (Figure 4), multiple proteins at several time points showed differential abundance, affecting multiple *N. tabacum* cellular pathways following INAP treatment.

## 4. Materials and Methods

### 4.1. Cell Suspension Cultures, INAP Treatment and Experimental Design

*N. tabacum* cell suspensions, sourced from the biochemistry department at the University of Johannesburg, were cultivated as previously described [8]. Three days post-subculture (mid-logarithmic stage), cells were induced with 1 mM INAP in accordance with Madala et al. [7]. The experimental design was comprised of control (C) and treated (T) samples for the selected time intervals of 0, 8, 16 and 24 h. Non-treated cells were also harvested at the specified time points as additional controls to monitor INAP-induced changes to the proteome (T8, T16, T24) against the background of normal growth processes and not related to the treatment (C8, C16, C24). Upon completion of the treatment time periods, the medium was filtered off using a vacuum-assisted filtration system. Cells harvested from multiple flasks were combined and divided into 0.5 g aliquots for replicate analyses. All samples collected (T and C) were subjected to equal conditions with the only variable consisting of the INAP treatment. Cells were flash frozen in liquid nitrogen to quench metabolic activity and prevent protein degradation. The experimental design included three independent biological repeats.

### 4.2. Protein Extraction and Two-Dimensional Gel Electrophoresis

The total protein extraction of the cell suspensions was performed following the trichloroacetic acid/acetone and phenol procedure [64]. Protein quantification was performed using the Amido Black method [65]. Two-DE was performed as previously described by Khoza et al. [65], as a prerequisite confirmation of differential protein expression in response to INAP treatment, prior to further in-depth iTRAQ analysis. In short, 140 µL samples were prepared using 100 μg of protein in 2 μL of 50% dithiothreitol (DTT) (*w*/*v*), 1.25 μL of ampholyte solution pH 3–10 (BioRad, Hercules, CA, USA) and the necessary amount of isoelectric focusing (IEF) buffer (containing 0.1% bromophenol blue) to make up the volume. The total sample volume was pipetted into a rehydration tray lane, upon which the IPG strip (BioRad, Hercules, CA, USA) selected for the narrow range (pH 4–7) was placed gel-side-down onto the sample. Each sample-containing lane was then covered with a layer of mineral oil and left overnight at room temperature. Following rehydration, the strips were removed, rinsed and transferred gel-side-up to the loading tray of the IPGphorII electrophoresis unit (Ettan^TM^ IPGphor II^TM^, GE Healthcare, Chicago, IL, USA) in the correct orientation. Wetted filter wicks were placed on either end of each of the IPG strips overlapping a small portion of the gel, and the electrodes were fitted to the strips. Following the covering of the strips with mineral oil, electrophoresis was commenced using the following programme settings: 250 V for 15 min, 4000 V for 1 h, 4000 V for 12,000 V h, and a 750 V holding step for 2 h. Once the IEF run had been completed, the IPG strips were removed and rinsed in 1X tank buffer followed by equilibration using two steps prior to electrophoresis in the second dimension. Here, the first step required the submergence of the strips in SDS equilibration buffer (6 M urea, 30% glycerol, 2% SDS, 50 mM Tris and trace bromophenol blue) containing 0.08 mg of DTT per 4 mL of buffer for 10 min at room temperature under constant agitation, after which the strips were rinsed in 1X tank buffer. The strips were then submerged in sodium dodecyl sulphate (SDS) equilibration buffer containing 0.1 mg of iodoacetamide per 4 mL of buffer for the second equilibration step of 10 min under constant agitation and subsequently washed using 1X tank buffer. Next, the strips were transferred to 10% SDS-polyacrylamide (PAGE) gels without the stacking gel. The strips were placed on the resolving gel, noting the direction of the pH range, and filter paper was loaded with 5 μL of marker and placed next to the strip. Prior to electrophoresis of the gels, the cassette was sealed with 2% agarose containing trace amounts of bromophenol blue. Once complete, the gels were removed from the cassettes, fixed and stained with Coomassie R-250 [66].

### 4.3. Protein Extraction for iTRAQ

A full set of samples representing the individual time points were ground with liquid nitrogen to form a fine powder before being transferred to pre-weighed plastic centrifuge tubes containing 3 mL of extraction buffer (6 M guanidine hydrochloride (CalBiochem, USA), 25 mM 4-(2-hydroxyethyl)piperazine-1-ethanesulfonic acid (HEPES), 5 mM dithiothreitol (DTT) and cOmplete™ Mini EDTA-free Protease Inhibitor Cocktail (Roche, Germany), pH 7.5). The tubes were weighed to determine the amount of starting material (±0.5 g), and 5% (*w*/*w*) insoluble polyvinylpyrrolidone (PVP) was added to the respective tubes. The solution was vortexed to ensure thorough mixing before being centrifuged at 9000× *g* for 10 min. Following centrifugation, the supernatants were transferred to sterile 50 mL Falcon tubes containing 20 mL of pre-chilled 98% analytical-grade acetone for overnight precipitation at −20 °C. Subsequently, the samples were centrifuged at 5000× *g* for 30 min and the supernatants were discarded. The resulting protein pellets were washed 3 times with 98% acetone under the same centrifugation conditions. For protein determination, the final pellet was resuspended in resuspension buffer (50 mM ammonium bicarbonate, 1% RapiGest™ SF buffer (Waters, USA) and 2.5 mM DTT at pH 8.5). The samples were quantified utilising the Amido Black assay [65]. The procedure was repeated for another full set of samples with the introduction of a 30 µg/mL lysozyme as an internal standard (≥90% Merck/Sigma, USA) ‘spike’ into the extraction buffer prior to the addition of the ground samples, and the protocol was completed to the final pelleting. Subsequent to the final centrifugation, the pellets were allowed to air-dry for the removal of excess acetone, and then the tubes were filled with nitrogen gas, sealed and sent to the Centre for Proteomic & Genomic Research (CPGR), University of Cape Town, South Africa, for 8-plex iTRAQ/nano LC/MS analysis.

### 4.4. Sample Preparation and Labelling for 8-plex iTRAQ

Beta-casein (98%, Merck/Sigma, USA) was added to each sample as the internal control (prior to digestion and labelling) at 6 pmoles. The sample volumes were adjusted to 10 µL with 50 mM triethylammonium bicarbonate buffer (TEAB) followed by reduction with 1 µL of 100 mM tris (2-carboxyethyl) phosphine (TCEP) in 50 mM TEAB and incubation at 60 °C for 1 h. The samples were then cooled to room temperature and alkylation of the cysteine residues achieved using 1 µL of methyl-methanethiosulfonate (MMTS, Sigma, USA, 5 mM final concentration) and an incubation of 30 min at room temperature. Next, the samples were diluted to 45 µL using 50 mM TEAB prior to the addition of 5 µL of mass spectrometry (MS)-grade trypsin (Promega, USA) and an overnight incubation at 37 °C.

Prior to labelling, evaluation of the tryptic digestion was performed using reverse-phase liquid chromatography. Sample volumes were reduced to 10 µL in a centrifugal rotary-evaporator (LabConco, USA) and 10 µL of 600 mM TEAB was added to each sample to a final concentration of 300 mM with the pH confirmed above 7.5. The iTRAQ reagents were prepared in accordance with the manufacturer’s (AB Sciex, USA) instructions. The contents of the vials were added to the respective sample (isobaric tags: 0 h T–113, 8 h C–114, 8 h T–115, 16 h C–116, 16 h T–117, 24 h C–118 and 24 h T–119), vortexed, centrifuged and incubated for 2 h at RT. One-microliter aliquots were taken from each sample and combined for desalting by double dilution with the loading solvent (2% acetonitrile in water, 15% trifluoroacetic acid) and the use of C18 ZipTips (Millipore, USA) as per the manufacturer’s instructions.

### 4.5. OFFGEL Electrophoresis

The samples were subjected to pI-based peptide separation using the 3100 OFFGEL fractionator and OFFGEL kit pH 3–10 (Agilent Technologies, USA) for 12 wells in accordance with the supplier’s protocol. Forty microliters of focusing buffer per well was used to rehydrate linear-gradient 13-cm-long IPG gel strips 10 min prior to sample loading. For sample loading, approximately 90 µg of sample was diluted with focusing buffer for a final volume of 1.8 mL and 150 µL of sample loaded in each well. Peptide focusing commenced up to a total of 20 kVh, 8000 V, and 50 µA.

### 4.6. LC MS/MS Analysis

LC/MS analysis was performed using the Q-Exactive Quadrupole-Orbitrap mass spectrometer (ThermoFisher Scientific, Waltham, MA, USA) coupled to a Dionex Ultimate 3000 nano-HPLC system. The samples were loaded onto C18 trap columns (Merck/Sigma, Rahway, NJ, USA) (100 µm × 20 mm × 5 µm) and chromatographic separation was performed utilising an Acclaim Pepmap C18 column (ThermoFisher Scientific, USA) (75 µm × 250 mm × 3 µm). Solvent A: 0.1% formic acid and solvent B: 80% acetonitrile/0.1% formic acid were used in a multi-step gradient for a time change of 78 min and a gradient change of 6–25%, generated at a flow rate of 250 nL/min with the mass spectrometer set to positive ionisation mode and a capillary temperature of 250 °C and 320 °C, respectively, with the applied electrospray voltage at 1.95 kV. The data acquisition was set up for a scan range of 350–2000 *m/z* for 100 ms at a 70,000 (@ *m/z* 200) resolution. Multiple charge states were used for MS/MS precursor selection at the same range for 50 ms at a 17,500 (@ *m/z* 200) resolution.

### 4.7. Data Analysis and Database Searching

All raw data obtained were subjected to searching and processing with Scaffold Q+ ver. 4.3.4. and 4.4.8 software (ProteomeSoftware, Portland, OR, USA). All acquired intensities for quantitation, accomplished by a spectral count, were normalised across all acquisition runs using medians for the multiplicatively normalised data. In order to narrow down the protein number and focus on the most significant proteins, the screening criteria of differential proteins were as follows: a fold change greater than 1.5 (either positive or negative) and a *p* value <0.05. Additionally, the intensity of each identified peptide was normalised for the respective protein. Subsequently, the reference channel was then normalised to generate a 1:1 fold change. For comparative analysis across time points, a threshold of 1.5-fold of the set normalised intensity values (NIVs), reported as median/density chart values, was selected for the determination of proteins flagged as undergoing differential changes when compared to the labelled 0 h T sample.

Analysis of all MS/MS sample data was performed using Mascot (Matrix Science, UK; version 2.4.1) and X! Tandem (The Global Proteome Machine (GPM), version CYCLONE (2010.12.01.1)). With trypsin selected as the digestive enzyme, the software was set up to search the UniprotSolan_2014120 database and the ArabRef3AUP000006548_290216 Uniprot (www.uniprot.org) sourced reference proteome for the *N. tabacum* samples. Searching of the databases was performed with a parent ion tolerance of 20 ppm and a fragment ion mass tolerance of 0.020 Da. The methylthio-derivative of cysteine and iTRAQ8plex of lysine along with the N-terminus were set as fixed modifications for both Mascot and X! Tandem. Variable modifications for Mascot included deamination of asparagine and glutamine, oxidation of methionine and iTRAQ8plex of tyrosine. The X! Tandem variable modifications listed were inclusive of those for Mascot as well as Glu->pyro-Glu of the N-terminus, ammonia loss of the N-terminus and gln->pyro-Glu of the N-terminus.

### 4.8. Data Analysis: Protein Identification Criteria and Bioinformatics Validation

Validation of the MS/MS peptide and protein identifications was achieved utilising Scaffold software (version Scaffold_4.3.4/4.4.0 Proteome Software Inc., USA). Acceptance of peptide identifications had to meet > 5.0% probability in order to achieve a false detection rate (FDR) of <0.5% by the Scaffold Local FDR algorithm. For acceptable protein identification, we adhered to a >98.0% probability to achieve an FDR < 1.0% containing a minimum of 2 identified peptides. Protein probabilities were assigned using the Protein Prophet algorithm [67], and proteins containing similar peptides that could not be differentiated by MS/MS alone were grouped together to satisfy parsimony principles [68].

Scaffold software was used for the iTRAQ analysis of the data sets. Following the launch of the Q+ quantitation browser, sample organisation was conducted in compliance with the label allocations (quant 1 for the 113-labelled sample, quant 2 for the 114-labelled sample, etc.) and completed for all samples within the data set. In subsequent data analyses, the 113-labelled sample (quant 1) was set as the reference sample (0 h T) to which all samples were compared. For comparative analysis, a threshold of 1.5-fold (±0.6 in Scaffold) of the set NIVs, reported as median/density chart values in Scaffold, and Log_2_ Fold values were selected for the determination of proteins displaying differential up/down-regulation. Thereafter, the data were further processed and, with protein sequence coverage consideration, a value of ≥0.5 was set to determine protein significance when the control was set to zero (0).

Information related to the proteins was obtained from the Uniprot database (www.uniprot.org; accessed on 1 September 2021 onwards). The protein sequences were retrieved and subjected to a TBLASTN (www.ncbi.nlm.nih.gov; accessed on 1 September 2021 onwards) search specific to the genome of *N. tabacum* (*Nicotiana tabacum* (taxid:4097)). The criteria utilised for the acceptance of associated genes were set using the lowest possible e-value with a minimum value of E-100, 90% coverage, 90% identity and the maximum score available for the selection of the best accession number. Following the search, validation of the identified genes was completed using related studies where possible, such as [8]. Classification of the proteins into the appropriate functional categories, namely Defence, Biosynthesis, Transport, DNA and Transcription, Metabolism and Energy, Translation, Unclassified, Response, Growth and Signalling, was achieved through the use of Uniprot classification, gene ontology classification and the available literature on the identifiable proteins within the data sets. Subsequently, the proteins were divided according to subcellular location, obtained from Uniprot, to either adaptation-related proteins composed of those from the cytosol, endoplasmic reticulum, Golgi apparatus, plasma membrane, nucleosome, vacuole, peroxisome, endosome and extracellular region or basic functionality proteins constituting the chloroplastic, mitochondrial and ribosomal proteins.

## 5. Conclusions

The two-dimensional electrophoretic and iTRAQ-based analysis of the proteome of *N. tabacum* cells in response to INAP revealed dynamic changes occurring over a 24-h period of investigation. In general, the responses were broad-based across several functional categories of proteins and indicative of aspects of plant defence responses as attained through pathogen exposure but focused on priming for increased defence preparedness. These included the up-regulation of proteins relating to processes such as cell wall modifications, ROS production, defence compound production and chromatin modulation, all of which are associated with responses triggered by effective priming agents. Moreover, the observed down-regulation of proteins involved in processes such as growth and lipid metabolism added additional support to the concept of a triggered defence-related response involving the redirection of cellular metabolic pathways following exposure to INAP. The dynamics of the response indicate that a form of homeostasis is re-established following the initial perturbation as previously reviewed by Kosová et al. [69,70]. In addition, the correlation of the obtained results to those from previous studies further reinforces the notion of INAP as a priming agent and contributes to a better understanding of its mechanism of action.

## Figures and Tables

**Table 1:** Processed data of the differentially regulated proteins of abundance observed in the INAP-treated *N. tabacum* cells *.

Protein Name	Accession Number	0 h (T)	8 h (T)	16 h (T)	24 h (T)	Gene Accession Number	Gene Name
**Biosynthesis**							
UDP-glucose:protein transglucosylase-like protein SlUPTG1 ^1^	Q6IV07_SOLLC	0.0	0.2	−0.3	0.0	XM 016639837.1 ^a^	Alpha-1,4-glucan-protein synthase
Uncharacterised protein ^2^	M1C547_SOLTU	0.0	0.7	0.3	0.5	XM 016633077.1 ^a^	Isopentenyl-diphosphate delta-isomerase I
Uncharacterised protein ^2^	M1C5Y4_SOLTU	0.0	1.2	−0.3	0.4	NM 001326080.1	UDP-glucuronic acid decarboxylase 6-like
Uncharacterised protein ^1^	K4BVZ4_SOLLC	0.0	−0.2	−0.6	−0.3	XM 016647169.1 ^b^	Dolichyl-diphosphooligosaccharide-protein glycosyltrasnferase
Uncharacterised protein ^1^	K4CET6_SOLLC (+1)	0.0	0.2	−0.8	−0.3	XM 016657513.1 ^b^	Long chain acyl-CoA synthetase 8-like
Cystathionine gamma-synthase ^3^	Q9ZPL5_TOBAC	0.0	0.0	−0.6	−0.4	AF097180.1	Alpha-1,4-glucan-protein synthase
**Defence**							
Annexin ^3^	Q9XEN8_TOBAC	0.0	0.6	0.4	0.4	NM 001325957.1	
Putative hydroxycinnamoyl transferase ^5^	B5LAV0_CAPAN (+5)	0.0	0.5	−0.1	0.3	NM 001325623.1	Shikimate O-hydroxy-cinnamoyltransferase
Superoxide dismutase (Cu-Zn) ^8^	SODC_NICPL	0.0	−0.4	−0.8	−0.4	XM 016594235.1 ^a^	
Polygalacturonase inhibiting protein ^4^	C4PG28_9SOLN	0.0	−0.3	−0.6	−0.3	XM 016650938.1 ^a^	Polygalacturonase inhibitor-like
Uncharacterised protein ^1^	K4ASK1_SOLLC	0.0	0.1	−0.9	−0.3	XM 016578137.1 ^a^	Callose synthase 9-like
**DNA and Transcription**							
Uncharacterised protein ^1^	K4CMV1_SOLLC	0.0	−0.6	−0.7	−0.9	XR 001654730.1 ^b^	Replication protein A 70kDa DNA-binding
						XM 016640989.1 ^b^	
Endonuclease 1 ^1^	G3XKQ7_SOLLC (+2)	0.0	−0.2	−0.6	−0.5	XM 016636178.1 ^a^	
Histone H2B ^6^	A2IBL2_NICBE	0.0	0.1	−0.6	−0.2		
Histone H2B ^3^	H2B_TOBAC	0.0	−0.4	−0.7	−0.2		
**Growth**							
Uncharacterised protein ^1^	K4BLX1_SOLLC	0.0	0.0	−0.7	−0.4	XM 016647000.1 ^b^	BONZAI 1-like
						XM 016649340.1 ^b^	
DWARF1/DIMINUTO ^1^	Q66YT8_SOLLC	0.0	−0.2	−0.7	−0.4	XM 016612143.1 ^a^	Delta(24)-sterol reductase-like
Uncharacterised protein ^1^	K4BGV0_SOLLC	0.0	0.1	0.7	0.0	XM 016614569.1 ^b^	CDK5RAP3-like protein
**Metabolism and Energy**							
Uncharacterised protein ^1^	K4BDG0_SOLLC (+2)	0.0	−0.1	−1.3	−0.7	XM 016594393.1 ^b^	Very-long-chain 3-oxoacyl-CoA reductase 1-like
						XM 016626206.1 ^b^	
Uncharacterised protein ^2^	M1A251_SOLTU	0.0	−0.4	−0.8	−0.5	XM 016617189.1 ^b^	Suberization-associated anionic peroxidase 1-like
Probable pectate lyase P18 ^1^	K4BDF4_SOLLC	0.0	0.1	−0.2	0.0	XM 016621815.1 ^b^	
Uncharacterised protein ^1^	K4AT35_SOLLC (+2)	0.0	−0.2	0.0	0.6	XM 0166417471 ^a^	26S Proteasome non-ATPase
						XM 016629739.1 ^a^	
Glycylpeptide N-tetradecanoyltransferase ^2^	M0ZRQ4_SOLTU	0.0	0.5	0.1	0.4	XM 016628510.1 ^a^	
Methylenetetrahydro-folate reductase ^1^	K4C2P8_SOLLC (+2)	0.0	0.7	0.4	0.5	XM 016660349.1 ^a^	
						XM 016660348.1 ^a^ XM 016660347.1 ^a^	
**Response**							
Glycine-rich RNA-binding protein ^3^	B2YKT9_TOBAC	0.0	0.1	0.4	0.6		
Putative methyltransferase ^2^	A4UV20_SOLTU (+3)	0.0	−0.3	−0.8	−0.4	NM 001324795.1	
						LC052785.1	
Uncharacterised protein ^2^	M1C4X5_SOLTU	0.0	−0.3	−0.9	−0.9	XM 016650151.1 ^b^ XM 016650150.1 ^b^	Probable methyltransferase
**Signalling**							
Ras-related GTP-binding protein ^3^	Q40569_TOBAC	0.0	0.1	−0.7	−0.4	NM 001325922.1 X72212.1	
Uncharacterised protein ^2^	M1B9Y5_SOLTU	0.0	0.0	−0.7	−0.3	XM 016620909.1 ^b^	Putative receptor kinase
Calcium-dependent protein kinase ^3^	O81390_TOBAC	0.0	−0.5	−0.7	−0.5	NM 001324640.1 AF072908.1	
Beta-tubulin ^9^	Q676U1_NICAT	0.0	0.6	0.2	0.2	NM 001325510.1	
						KP316400.1	
Alpha subunit of SnRK1 ^10^	M1LH79_9SOLN	0.0	0.8	0.1	0.2	XM 016633060.1 ^a^	
Phospholipase D ^1^	K4BAK2_SOLLC	0.0	0.6	−1.1	−0.6	XM 016601192.1 ^b^	Ras-related protein RAB8-1
**Translation**							
Peptidyl-prolyl cis-trans isomerase ^5^	B1PDK0_CAPAN	0.0	0.4	0.6	0.2	XM 016614927.1 ^b^	
**Transport**							
Clathrin heavy chain ^1^	K4C1T2_SOLLC	0.0	−0.2	−0.6	−0.5	XM 016641028.1 ^a^	Clathrin heavy chain 1
Plasma membrane ATPase 4 ^8^	PMA4_NICPL	0.0	0.0	−0.7	−0.4	XM 016619330.1 ^a^	
Uncharacterised protein ^2^	M1D6E0_SOLTU	0.0	−0.1	−0.7	−0.4	XM 016602228.1 ^a^	ATPase 2
Pleiotropic drug resistance protein 1 ^8^	PDR1_NICPL	0.0	−0.2	−1.1	−0.5	XM 016622655.1 ^a^	
Clathrin heavy chain ^1^	K4C5S4_SOLLC	0.0	−0.3	−0.8	−0.8	XM 016613710.1 ^a^	
Reticulon-like protein ^2^	M0ZGB8_SOLTU	0.0	0.1	−0.8	0.0	XM 016611959.1 ^b^	
Uncharacterised protein ^1^	K4CIM3_SOLLC (+2)	0.0	−0.4	−0.9	−1.0	XM 016622951.1 ^b^	Protein transport protein SEC16B
**Basic Functionality Proteins**							
Glycine dehydrogenase (decarboxylating) ^2^	GCSP_SOLTU (+2)	0.0	0.4	0.5	0.5	XM 016601634.1 ^a^	
Aspartate aminotransferase ^1^	K4CG60_SOLLC	0.0	0.5	0.5	0.6	XM 016642299.1 ^a^	
Uncharacterised protein ^1^	K4C412_SOLLC	0.0	−0.2	−0.4	−0.6	XM 016617152.1 ^a^	Fumarate hydratase 1
Tobacco pre-pro-cysteine proteinase^3^	Q43579_TOBAC (+2)	0.0	0.1	0.0	0.3	Z13959.1	
Cytochrome c oxidase subunit 2 ^3^	Q5MA02_TOBAC	0.0	0.1	−0.7	−0.5		
Mitochondrial small heat shock protein ^5^	D9IAX1_CAPAN (+1)	0.0	−0.1	−0.6	0.0		
Uncharacterised protein ^2^	M0ZUV5_SOLTU	0.0	−0.2	−0.8	−0.4	XM 016634781.1 ^b^	Cytochrome P450

* The identified proteins are listed according to the Uniprot (www.uniprot.org; accessed 1 September 2021 onwards) functional categories, including the identified TBLASTN (www.ncbi.nlm.nih.gov; accessed 1 September 2021 onwards) genes associated with the respective proteins in accordance with the data analysis and bioinformatics validation. For the values in cells, green and pink indicate up- and down-regulation, respectively. For comparative analysis across time points, the threshold of 1.5-fold of the set normalised intensity values (NIVs) and the reported Log_2_ fold values (R) (Tables S1 and S2) were processed as a 0.5 value difference, with protein sequence coverage consideration, for determination of protein significances when compared to the labelled 0 h T sample set to 0. ^a^ Predicted or putative proteins, ^b^ 70% Identity gene hit proteins ^1^ OS, *Solanum lycopersicum*; ^2^ OS, *Solanum tuberosum*; ^3^ OS, *Nicotiana tabacum*; ^4^ OS, *Solanum torvum*; ^5^ OS, *Capsicum annuum*; ^6^ OS, *Nicotiana benthamiana*; ^7^ OS, *Capsicum chinense*; ^8^ OS, *Nicotiana plumbaginifolia*; ^9^ OS, *Nicotiana attenuata*; ^10^ OS, *Solanum berthaultii.*

## Data Availability

Data are available within the article or its supplementary materials. Data are available on request from the author (for data set identifier, username and password) for repository JPST002055/PXD040325. Repository citation: Okuda, S. et al. jPOSTrepo: an international standard data repository for proteomes. Nucl. Acids Res. 45 (D1): D1107-D1111 (2017). doi:10.1093/nar/gkw1080.

## Supplementary Materials

The following supporting information can be downloaded at: https://www.mdpi.com/article/10.3390/plants12051137/s1, Table S1: Identified adaptation-related proteins from INAP-treated *Nicotiana tabacum* cells. Proteins listed are from the following cellular sub-locations: cytosol, endoplasmic reticulum, Golgi apparatus, plasma membrane, nucleosome, vacuole, peroxisome, endosome and extracellular region; Table S2: Identified basic functionality proteins in INAP-treated *Nicotiana tabacum* cell suspensions. Proteins listed are from the following cellular sub-locations: chloroplast, mitochondria and ribosome; Table S3: Raw data list of all proteins identified through iTRAQ before implementations of the threshold for significant protein identification.
